# Trends in Suspected Fentanyl-Involved Nonfatal Overdose Emergency Department Visits, by Age Group, Sex, and Race and Ethnicity — United States, October 2020–March 2024

**DOI:** 10.15585/mmwr.mm7416a2

**Published:** 2025-05-08

**Authors:** Cassandra M. Pickens, Joohyun Park, Shannon M. Casillas, Stephen Liu, Michael Sheppard, Erin K. Stokes, Jean Y. Ko, Seung Hee Lee

**Affiliations:** ^1^Division of Overdose Prevention, National Center for Injury Prevention, CDC; ^2^Detect and Monitor Division, Office of Public Health Data, Surveillance, and Technology, CDC.

**Summary**

**What is already known about this topic?**

Overdose deaths involving synthetic opioids including fentanyl increased during the past decade, with declines beginning in mid-2023. Data on nonfatal overdoses involving fentanyl are limited.

**What is added by this report?**

Fentanyl-involved nonfatal overdose emergency department (ED) visit rates increased in a majority of demographic groups from late 2020 through mid-2023, with highest rates and largest increases among non-Hispanic American Indian or Alaska Native persons. Overall rates increased 8.7% per quarter from quarter (Q) 4 2020 to Q3 2023, then declined 11.0% per quarter from Q3 2023 to Q1 2024.

**What are the implications for public health practice?**

Despite recent declining trends, fentanyl-involved nonfatal overdose ED visits remain high (a rate of 2.9 per 10,000 ED visits in Q1 2024, versus 1.4 in Q4 2020). ED interventions to increase naloxone access and availability and linkage to and retention in evidence-based care of persons who have experienced an overdose could reduce future nonfatal and fatal overdoses.

## Abstract

Fatal overdoses involving synthetic opioids such as fentanyl increased sharply during the past decade. Recent data indicate declines in deaths with illegally manufactured fentanyls detected beginning in mid-2023. However, timely data on nonfatal overdoses involving fentanyl are limited. Emergency department (ED) data from CDC’s National Syndromic Surveillance Program during October 2020–March 2024 were analyzed. Quarterly trends in rates of suspected nonfatal overdose of unintentional or undetermined intent involving fentanyl or fentanyl analogs (fentanyl-involved nonfatal overdoses) (i.e., the number of ED visits for fentanyl-involved nonfatal overdose per 10,000 total ED visits) were analyzed overall and by age group, sex, and race and ethnicity. During quarter (Q) 4 (October–December) 2020 to Q3 (July–September) 2023, rates of fentanyl-involved nonfatal overdose ED visits increased 8.7% per quarter, from 1.4 to 3.5 per 10,000 ED visits, then declined 11.0% per quarter, to 2.9 per 10,000 ED visits, from Q3 2023 to Q1 (January–March) 2024. Trends increased among a majority of demographic groups through mid-2023, with the highest rates and the largest increases among non-Hispanic American Indian or Alaska Native persons (e.g., 11.9 per 10,000 ED visits in Q3 2023, and an average quarterly increase of 9.0%, respectively). Providers in EDs have an important role in preventing fentanyl-involved nonfatal overdoses. Buprenorphine, a medication used to treat opioid use disorder that can be initiated in an ED, might benefit persons who use EDs as a main source of medical care. In addition, comprehensive services, including screening and treatment of co-occurring mental health conditions, as well as evidence-based prevention, treatment, and recovery support services, might be initiated in EDs because these might be particularly important in communities at high risk for fentanyl overdoses.

## Introduction

Drug overdose remains a substantial public health concern in the United States. Overdose deaths involving synthetic opioids such as fentanyl have increased substantially during the past decade (*1*). However, more recent data indicate that, beginning in mid-2023, deaths with illegally manufactured fentanyl and fentanyl analogs detected have declined (*2*). Less is known about trends in fentanyl-involved nonfatal overdoses. Persons who experience a nonfatal overdose are more likely to experience a future fatal overdose (*3*); therefore, identifying populations affected by fentanyl-involved nonfatal overdoses might provide information that could guide prevention strategies and enhance recovery support. A study examining discharge diagnosis codes in emergency department (ED) data found increases in synthetic opioid-involved nonfatal overdoses, primarily driven by fentanyl, during October 2019–September 2021 (*4*). Using more recent ED visit data, including the reported chief complaint field, CDC identified trends in suspected* nonfatal overdose of unintentional or undetermined intent involving fentanyl or fentanyl analogs (fentanyl-involved nonfatal overdose), overall and by patient demographic characteristics.

## Methods

### Data Source

Fentanyl-involved nonfatal overdose ED visits were identified using data from CDC’s National Syndromic Surveillance Program (NSSP), which provides near real-time electronic health record data on ED visits, often within 24–48 hours. NSSP involves collaboration among state and local health departments, CDC, and other partners, and currently includes 80% of U.S. EDs.^†^ ED visits for fentanyl-involved nonfatal overdoses were queried using CDC’s fentanyl overdose syndrome definition,^§^ which identifies visits using free-text chief complaint fields and discharge diagnosis codes. Data from Q4 (October–December) 2020 through Q1 (January–March) 2024 were queried.

### Data Classification and Inclusion and Exclusion Criteria

The analysis was restricted to 3,056 of 4,969 (62%) U.S. EDs reporting consistent and complete data during the study period.^¶^ Counts of fentanyl-involved nonfatal overdoses and total ED visits for any cause were aggregated quarterly, overall and by age group, sex, and race and ethnicity. Persons aged 0–14 years were excluded from the age-specific analysis because of data suppression criteria (fewer than 20 cases per quarter). Persons of Hispanic or Latino (Hispanic) ethnicity, irrespective of race, were classified as Hispanic. For the remaining categories, non-Hispanic persons were reported by their indicated single race classification (i.e., American Indian or Alaska Native [AI/AN], Black or African American [Black], White, or other race). Asian, multiple race, other race, and Native Hawaiian or Pacific Islander persons were aggregated into the other race category because of small counts. Although a majority of the race and ethnicity data in NSSP are likely based on self-report, reporting source is not indicated. Visits with missing or unknown values for certain variables were excluded from analyses of that variable.

### Data Analysis

Quarterly rates of fentanyl-involved nonfatal overdose ED visits (fentanyl-involved nonfatal overdoses per 10,000 total ED visits of any cause) and average quarterly percent change (AQPC) in rates, were analyzed overall and by age group, sex, and race and ethnicity. Analyses were conducted using Joinpoint regression (version 5.0.2; National Cancer Institute). Quarterly percent change (QPC) estimates for trend segments were calculated when trend changes were identified. P-values <0.05 were considered statistically significant. This activity was reviewed by CDC, deemed not research, and was conducted consistent with applicable federal law and CDC policy.**

## Results

### Overall Trends in Fentanyl-Involved Nonfatal Overdoses

During October 2020–March 2024, a total of 86,404 fentanyl-involved nonfatal overdose ED visits were identified. The rate of ED visits for fentanyl-involved nonfatal overdoses increased an average of 5.4% per quarter (Table). The overall rate increased from 1.4 (Q4 2020) to 2.9 (Q1 2024) per 10,000 ED visits and peaked in Q3 2023 at 3.5 per 10,000 ED visits (Figure). From Q4 2020 to Q3 2023, rates increased at a constant rate of 8.7% per quarter, subsequently declining 11.0% per quarter, from Q3 2023 to Q1 2024.

**TABLE:** Trends* in rates of suspected fentanyl-involved nonfatal overdose emergency department visits,^†^ overall and by demographic characteristics and quarter^§^ — United States, October 2020–March 2024

Characteristic	Average quarterly % change (95% CI)	Trend segments, quarterly % change (95% CI)		
		Segment 1	Segment 2	Segment 3
**Overall**	5.4 (4.4 to 6.5)^¶^	Q4 2020–Q3 2023 8.7 (7.9 to 10.1)^¶^	Q3 2023–Q1 2024 −11.0 (−16.9 to −2.8)^¶^	NA
Age group, yrs**				
15–24	3.3 (2.0 to 4.7)^¶^	Q4 2020–Q4 2021 2.1 (−7.5 to 7.2)	Q4 2021–Q2 2023 9.8 (7.4 to 16.4)^¶^	Q2 2023–Q1 2024 −7.1 (−14.0 to −2.2)^¶^
25–34	5.5 (4.5 to 6.5)^¶^	Q4 2020–Q2 2023 9.3 (8.3 to 10.8)^¶^	Q2 2023–Q1 2024 −6.3 (−11.5 to −1.6)^¶^	NA
35–54	7.7 (6.9 to 8.7)^¶^	Q4 2020–Q2 2023 11.7 (10.6 to 13.3)^¶^	Q2 2023–Q1 2024 −4.6 (−8.9 to 0.1)	NA
≥55	6.7 (5.9 to 7.6)^¶^	Q4 2020–Q3 2023 9.8 (9.0 to 11.0)^¶^	Q3 2023–Q1 2024 −9.0 (−13.7 to −2.0)^¶^	NA
**Sex^††^**				
Female	5.4 (4.0 to 7.2)^¶^	Q4 2020–Q3 2023 8.5 (7.5 to 11.0)^¶^	Q3 2023–Q1 2024 −9.8 (−19.2 to 0.5)	NA
Male	5.4 (4.4 to 6.4)^¶^	Q4 2020–Q3 2023 8.7 (7.9 to 10.1)^¶^	Q3 2023–Q1 2024 −11.2 (−17.2 to −3.1)^¶^	NA
**Race and ethnicity^§§^**				
AI/AN	9.0 (7.3 to 12.7)^¶^	Q4 2020–Q3 2023 11.6 (10.3 to 25.5)^¶^	Q3 2023–Q1 2024 −4.5 (−14.6 to 8.3)	NA
Black or African American	6.9 (6.1 to 7.8)^¶^	Q4 2020–Q3 2023 10.2 (9.4 to 11.4)^¶^	Q3 2023–Q1 2024 −9.4 (−14.1 to −2.2)^¶^	NA
White	4.9 (3.9 to 5.9)^¶^	Q4 2020–Q2 2023 8.5 (7.4 to 10.1)^¶^	Q2 2023–Q1 2024 −6.5 (−12.6 to −1.8)^¶^	NA
Hispanic or Latino	3.9 (2.6 to 5.7)^¶^	Q4 2020–Q3 2023 6.8 (5.7 to 9.5)^¶^	Q3 2023–Q1 2024 −10.6 (−18.9 to 0.1)	NA
Other race^¶¶^	1.9 (0 to 3.8)	Q4 2020–Q1 2022 −2.2 (−15.1 to 3.8)	Q1 2022–Q2 2023 12.4 (8.1 to 23.7)^¶^	Q2 2023–Q1 2024 −7.3 (−20.0 to −0.5)^¶^

**Abbreviations:** AI/AN = American Indian or Alaska Native; ED = emergency department; NA = not applicable; NH = non-Hispanic; Q = quarter.

* Joinpoint Regression Program

^†^ Fentanyl-involved nonfatal overdoses per 10,000 ED visits.

^§^ Q1 = Jan–Mar; Q2 = Apr–Jun; Q3 = Jul–Sep; Q4 = Oct–Dec.

^¶^ The average quarterly percent change or quarterly percent change is significantly different from zero at p<0.05.

** Children and adolescents aged 0–14 years were excluded from the age-specific analysis because of low case count and suppression rules (i.e., fewer than 20 cases per quarter). Sample sizes (the number of total ED visits for any cause) by age group were as follows: 15–24 years, 41,237,765; 25–34 years, 49,743,218; 35–54 years, 83,551,045; and ≥55 years, 121,955,122.

^††^ Sample sizes (the number of total ED visits of any cause) were 189,880,424 for females and 157,665,771 for males.

^§§^ Persons of Hispanic or Latino (Hispanic) ethnicity, regardless of race, were classified as Hispanic. For the remaining categories, persons who were NH are reported by their indicated single race classification (e.g., AI/AN, Black or African American [Black], White, or other race). Several groups were aggregated into the other race category because of small counts (i.e., to avoid data suppression). The other race category includes NH Asian, NH Native Hawaiian or Pacific Islander, and NH multiple race or other race persons. Sample sizes (the number of total ED visits of any cause) by race and ethnicity were as follows: NH AI/AN, 2,319,488; NH Black, 60,789,083; NH White, 165,152,596; Hispanic, 50,915,083; and NH other race, 19,032,780.

^¶¶^ Sample sizes (the number of total ED visits for any cause among groups in the other race category were as follows: NH Asian, 4,885,812; NH Native Hawaiian or Pacific Islander, 642,315; and NH multiple or other race, 13,504,653.

**FIGURE:**
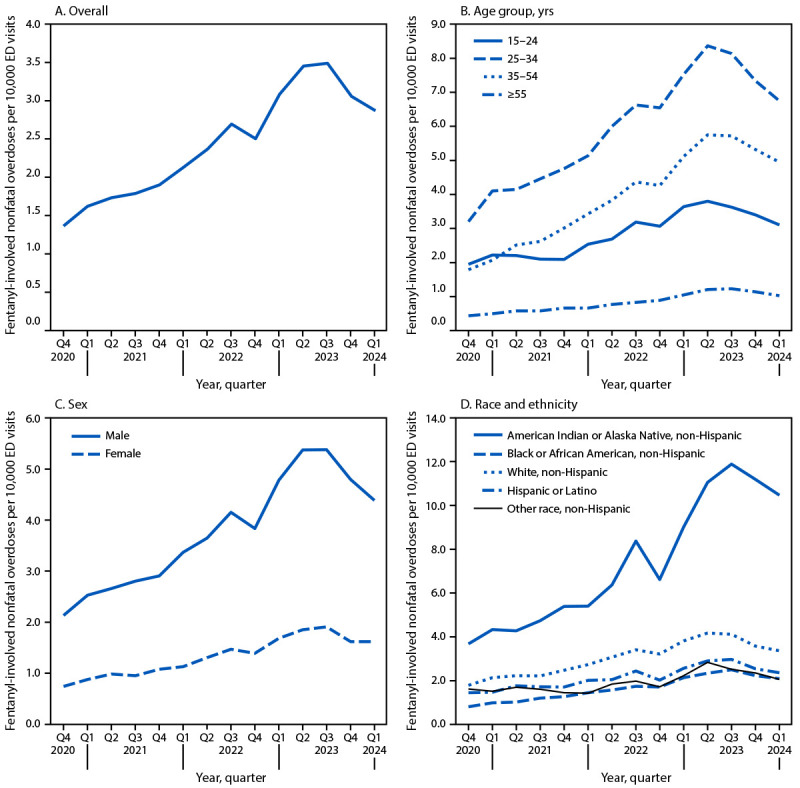
Quarterly rates* of suspected fentanyl-involved nonfatal overdose emergency department visits,^†,^^§^ overall and by demographic characteristics^¶^^,^** — United States, October 2020–March 2024 **Abbreviations:** AI/AN = American Indian or Alaska Native; ED = emergency department; Q = quarter. * The y-axis ranges are different in each panel. ^†^ Visits with missing or unknown values for certain variables were excluded from analyses of that variable only (e.g., visits with missing sex were excluded from the sex-specific analysis). ^§^ Q1 = Jan–Mar; Q2 = Apr–Jun; Q3 = Jul–Sep; Q4 = Oct–Dec. ^¶^ Children aged 0–14 years were excluded from the age-specific analysis because of low case counts and suppression rules (i.e., fewer than 20 cases per quarter). ** Persons of Hispanic or Latino (Hispanic) ethnicity, regardless of race, were classified as Hispanic. For the remaining categories, persons who were non-Hispanic are reported by their indicated single race classification (e.g., AI/AN, Black or African American, White, or other race). Several groups were aggregated into the other race category because of small counts (i.e., to avoid data suppression). The other race category includes non-Hispanic Asian, non-Hispanic Native Hawaiian or Pacific Islander, and non-Hispanic multiple race or other race persons.

### Age-Specific Trends

Fentanyl-involved nonfatal overdose rates were highest among adults aged 25–34 years and lowest among those aged ≥55 years (Figure). The AQPC ranged from 3.3% among persons aged 15–24 years to 7.7% among those aged 35–54 years (Table). Among adults aged ≥25 years, rates increased from Q4 2020 through mid-2023, with QPCs ranging from 9.3% to 11.7% among adults aged 25–34, 35–54, and ≥55 years. Among persons aged 15–24 years, rates remained stable from Q4 2020 through Q4 2021 and subsequently increased 9.8% per quarter from Q4 2021 to Q2 2023. Rates significantly declined from mid-2023 to Q1 2024 among those aged 15–24, 25–34, and ≥55 years.

### Sex-Specific Trends

Among males, fentanyl-involved nonfatal overdose rates increased from 2.1 to 4.4 per 10,000 ED visits during the study period, peaking at 5.4 per 10,000 ED visits in mid-2023 (Figure). In females, the rate increased from 0.7 per 10,000 ED visits in Q4 2020 to 1.6 in Q1 2024, with the highest rate of 1.9 occurring in mid-2023. The AQPC was similar among males and females (5.4%) (Table). Fentanyl-involved nonfatal overdose rates increased from Q4 2020 to Q3 2023 among both males (QPC = 8.7%) and females (QPC = 8.5%). Among males, this trend was followed by a quarterly decline of 11.2% from Q3 2023 to Q1 2024. The decline among females during this period was not statistically significant.

### Race- and Ethnicity-Specific Trends

Among racial and ethnic groups, the highest rate of fentanyl-involved nonfatal overdose ED visits (e.g., 11.9 per 10,000 visits in Q3 2023 (Figure), and the highest AQPC (9.0%) were among AI/AN persons (Table). Among all racial and ethnic groups (except persons in the combined other race category), statistically significant increasing trends were observed from Q4 2020 through mid-2023, with QPCs ranging from 6.8% among Hispanic persons to 11.6% among AI/AN persons. Among persons in the combined other race group, rates remained stable from Q4 2020 through Q1 2022 and increased by 12.4% quarterly from Q1 2022 to Q2 2023. From mid-2023 to Q1 2024, statistically significant QPCs were observed among those who were Black (9.4% decline), White (6.5% decline), and other races (7.3% decline); declines among AI/AN and Hispanic persons were not statistically significant.

## Discussion

From Q4 2020 through mid-2023, rates of fentanyl-involved nonfatal overdose ED visits increased. Rates subsequently declined through Q1 2024; the declines were observed overall and across a majority of demographic groups. These findings are consistent with recent mortality data, which indicate that overdose deaths with illegally manufactured fentanyl and fentanyl analogs detected peaked in mid-2023 and started to decline thereafter (*2*). The overall declines in both nonfatal and fatal overdoses involving fentanyl are encouraging; however, more time is needed to determine whether these observed decreases will be sustained and to identify factors responsible for the observed declines.

EDs are critical locations for implementing strategies that might prevent future or repeat fentanyl-involved overdoses among patients with substance use disorder, with a history of drug use, or who experienced a recent nonfatal overdose, particularly among those persons for whom an ED is the primary contact with the health care system. EDs can introduce overdose prevention strategies, initiate treatment, and link persons who have opioid use disorder to care. Initiating buprenorphine and other medications for opioid use disorder in EDs can offer a pathway to recovery by quickly stabilizing withdrawal symptoms and connecting patients to ongoing treatment.^††^ Other ED-based response strategies include providing naloxone to persons who recently experienced an overdose or to their families; naloxone reverses opioid overdose and can be used at home (*5*). Some recent data suggest that patient refusals of transport to an ED via emergency medical service (EMS) are increasing (*6*); training first responders (e.g., EMS personnel) and equipping them with naloxone and methods to link persons who experience overdoses to health care resources might also be important. Administration of naloxone can mean the difference between a nonfatal and fatal fentanyl-involved overdose. This dataset does not include information about the proportion of fentanyl-involved nonfatal overdose ED visits for which naloxone was administered or what proportion of visits were by persons who experienced a previous overdose. However, recent mortality data from 38 U.S. jurisdictions demonstrate that approximately two thirds (65.9%) of fatal overdoses (from any drug) in 2023 had at least one opportunity for intervention, such as having a potential bystander present (42.6%), a mental health diagnosis (28.7%), or a previous overdose (13.5%), whereas fewer than one quarter (23.7%) of fatal overdoses had documentation that naloxone was administered.^§§^

These findings highlight an urgent need to expand interventions, including naloxone distribution and training, as well as linkage to treatment services, which have the potential to not only reduce the likelihood of fatal overdoses but also to help prevent recurrent overdoses among those who survive. In addition, screening, treating, or referring patients for co-occurring mental health conditions can be done in an ED or any other setting, aligning with the Substance Abuse and Mental Health Services Administration’s “No Wrong Door” policy for treatment access, which states that effective systems must ensure that persons needing treatment will be identified, evaluated, and receive treatment, either directly or through appropriate referral, no matter where they seek services.^¶¶^ ED-based recovery support programs can include peer recovery specialists who share similar lived experience, such as treatment for addiction, to help facilitate linkage to care and recovery support services.***

This study found that the highest rates of fentanyl-involved nonfatal overdose ED visits were among younger adults aged 25–34 years, males, and AI/AN persons. Although information on fentanyl-involved nonfatal overdoses by demographic categories is limited, other drug overdose data such as EMS or mortality data can provide useful context. In a recent analysis using EMS encounter data, the highest rates of opioid-involved nonfatal EMS encounters were among males and adults aged 25–34 years (*6*). Moreover, in 2021 and 2022, the highest age-adjusted rates of all drug overdose mortality were among males (45.1 and 45.6 per 100,000 population, respectively) and AI/AN persons (56.6 and 65.2, respectively) (*1*). In the current study, the sharpest increase in rates of fentanyl-involved nonfatal overdoses was among AI/AN persons, similar to recent trends among all drug overdose deaths from 2021 to 2022, which increased 15.0% among this group (*1*). AI/AN communities are at increased risk for substance use related injury and harm (*7*). Tailored prevention measures might help reduce exposure to substance use.

### Limitations

The findings in this report are subject to at least six limitations. First, fentanyl-involved nonfatal overdoses might be underreported or misclassified because of hospital drug testing practices (*8*); however, some jurisdictions recently mandated testing for fentanyl as part of ED urine toxicology screens, which might improve ascertainment.^†††^ Second, the syndrome definition used in this study cannot distinguish between illegally manufactured fentanyl (or fentanyl analogs) and prescription fentanyl; however, a majority of fentanyl-involved overdose deaths are caused by illegally manufactured fentanyl (*2*). Third, ED data quality and completeness, including demographic data, vary by facility. Fourth, this dataset only captures nonfatal overdoses treated in EDs and might not represent persons who overdose in community settings and are not transported to an ED. Fifth, there could be a small number of fatal overdoses in this study; approximately 1% of fentanyl-involved overdose ED visits in this study were marked as ending in death. Finally, rates of fentanyl-involved nonfatal overdoses among AI/AN persons could be underestimated because of racial misclassification of AI/AN persons (*9*); further, data from a majority of tribal-specific health facilities are not included in NSSP.

### Implications for Public Health Practice

ED interventions to increase access to and availability of naloxone and to expand linkage to and retention in evidence-based care, including medications for opioid use disorder, are important. Focusing activities in communities with high or rising rates of fentanyl overdose, such as AI/AN communities, might help decrease both nonfatal and fatal overdoses. Ongoing monitoring of trends in fentanyl-involved nonfatal overdoses by state and local jurisdictions can identify areas in need of evidence-based prevention, treatment, and recovery support services.

## Footnotes

* The term suspected is used because discharge diagnosis codes (i.e., codes that indicate fentanyl-involved poisoning) might be preliminary or missing in syndromic surveillance data. ED visits are classified using chief complaint data and discharge diagnosis codes (the discharge diagnosis codes might be preliminary).

§
CDC Fentanyl Overdose v2 Parsed Technical Brief and Factsheet

¶ To reduce artifactual impact from changes in reporting patterns, analyses were restricted to facilities with more consistent reporting of more complete data (coefficient of variation ≤45% and average weekly informative discharge diagnosis ≥75% complete during the study period); How Data Quality Filters Work | National Syndromic Surveillance Program (NSSP) | CDC. A total of 3,056 facilities were included in this analysis (out of 4,969 ED facilities with at least one visit during this time). Fatal overdoses were not excluded but comprised approximately 1% of fentanyl-involved overdose ED visits in NSSP during the study period.

** 45 C.F.R. part 46.102(l)(2), 21 C.F.R. part 56; 42 U.S.C. Sect. 241(d); 5 U.S.C. Sect. 552a; 44 U.S.C. Sect. 3501 et seq.

††
Initiating Buprenorphine Treatment in the Emergency Department | National Institute on Drug Abuse (NIDA)

§§
SUDORS Dashboard: Fatal Drug Overdose Data | Overdose Prevention | CDC

¶¶
Screening and Treatment of Co-Occurring Disorders | SAMHSA

***
ED based substance use response toolkit

††† For example, in October 2023, Maryland mandated that fentanyl be added to toxicology screens (Legislation - SB0914). As of December 2023, Pennsylvania also mandated that fentanyl (and xylazine) be added to ED toxicology screens Senate Bill 683 Information; 2023-2024 Regular Session - The Official Website of the Pennsylvania General Assembly
